# BCL6 modulation of acute lymphoblastic leukemia response to chemotherapy

**DOI:** 10.18632/oncotarget.8273

**Published:** 2016-03-22

**Authors:** William L. Slone, Blake S. Moses, Ian Hare, Rebecca Evans, Debbie Piktel, Laura F. Gibson

**Affiliations:** ^1^ Alexander B. Osborn Hematopoietic Malignancy and Transplantation Program of The WVU Cancer Institute, Robert C. Byrd Health Sciences Center, West Virginia University School of Medicine, Morgantown, WV, USA; ^2^ Department of Microbiology, Immunology and Cell Biology, Robert C. Byrd Health Sciences Center, West Virginia University School of Medicine, Morgantown, WV, USA

**Keywords:** bone marrow microenvironment, acute lymphoblastic leukemia, BCL6, chemotherapy resistance

## Abstract

The bone marrow niche has a significant impact on acute lymphoblastic leukemia (ALL) cell phenotype. Of clinical relevance is the frequency with which quiescent leukemic cells, in this niche, survive treatment and contribute to relapse. This study suggests that marrow microenvironment regulation of BCL6 in ALL is one factor that may be involved in the transition between proliferative and quiescent states of ALL cells. Utilizing ALL cell lines, and primary patient tumor cells we observed that tumor cell BCL6 protein abundance is decreased in the presence of primary human bone marrow stromal cells (BMSC) and osteoblasts (HOB). Chemical inhibition, or shRNA knockdown, of BCL6 in ALL cells resulted in diminished ALL proliferation. As many chemotherapy regimens require tumor cell proliferation for optimal efficacy, we investigated the consequences of constitutive BCL6 expression in leukemic cells during co-culture with BMSC or HOB. Forced chronic expression of BCL6 during co-culture with BMSC or HOB sensitized the tumor to chemotherapy induced cell death. Combination treatment of caffeine, which increases BCL6 expression in ALL cells, with chemotherapy extended the event free survival of mice. These data suggest that BCL6 is one factor, modulated by microenvironment derived cues that may contribute to regulation of ALL therapeutic response.

## INTRODUCTION

Acute lymphoblastic leukemia (ALL) is the most common childhood malignancy. While two-thirds of cases present in children, the risk of ALL also increases with age in the adult population [1]. In both populations, relapse of disease is associated with poor prognosis, with relapsed disease often being more aggressive and refractory to treatment [2, 3]. Risk of relapse has been shown to be linked to the presence of refractory minimal residual disease (MRD) [4- 6]. The bone marrow is the most common site of ALL MRD, and consequently, the most common site of relapse [7]. Consistent with relapse in the bone marrow microenvironment (BMM), we and others have shown that bone marrow stromal cells (BMSC) and osteoblasts (HOB) provide protection to leukemic cells during chemotherapy treatment [8- 16]. However, the cell signaling pathways by which the BMM influences tumor cells to provide this protection remains incompletely understood.

While there are many diverse signaling pathways that converge on the phenotype of any tumor in response to microenvironment derived cues, the focus of the current investigation is on the modulation of ALL cell BCL6. BCL6 is a proto-oncogene that has been classically described in the setting of its influence on germinal center B-cells, as well as its role in the progression of diffuse large B-cell lymphoma [17- 28]. In these contexts, BCL6 has been well characterized as a regulator of B-cell proliferation, maturation, and resistance to DNA damage [29]. More recent work has highlighted the impact of BCL6 on immature and malignant hematopoietic cells. Increased expression of BCL6 in chronic myelogenous leukemia (CML) and acute lymphoblastic leukemia (ALL) has been shown to protect leukemic cells from chemotherapy induced DNA damage through the repression of p53 induced apoptosis [30, 31]. These studies, in addition to earlier work in germinal center biology, reflect the ability of BCL6 to influence leukemic cell phenotype through regulation of survival, differentiation, and cell cycle progression.

To address a fundamental gap that exists in understanding how the BMM impacts leukemic BCL6 we utilized the previously described *in vitro* model in which phase dim (PD) ALL cells migrate beneath BMSC or HOB and exhibit a chemotherapy-resistant phenotype. Our laboratory has previously characterized this dynamic *in vitro* model in which ALL cells seeded onto BMSC or HOB transiently migrate beneath the bone marrow stromal layer, generating the “phase dim” population [13, 15]. This population of ALL cells was characterized by quiescence and chemotherapy resistance while in this *in vitro* niche. However, removal from beneath the stromal layer results in a return to chemotherapy sensitivity [13]. Furthermore, this PD characteristic was specific to ALL cells co-cultured with BMSCs or HOBs, as PD populations, which readily migrated beneath co-cultures comprised of non-bone marrow derived adherent layers, were not protected from chemotherapy-induced death [13] suggesting the observed effect is not simply physical protection from cytotoxic drugs. Utilizing this co-culture model to represent BMM protected and resistant ALL cells we found that co-culture with BMSC or HOB reduced the abundance of tumor cell BCL6, coincident with increased survival and quiescence of a subset of tumor cells in contact with BMSC or HOB. Furthermore, chronic forced expression of BCL6 in this quiescent tumor cell population resulted in sensitization to chemotherapy. These observations suggest that the BMM influenced leukemic cell BCL6 protein abundance has the potential to contribute to the generation of a quiescent, drug resistant population of tumor cells and that strategies aimed at disruption of this pathway may prove to be an effective means by which to diminish MRD and relapse of ALL.

## RESULTS

### Co-culture with BMSC or HOB reduces BCL6 in ALL cells

**Figure 1:**
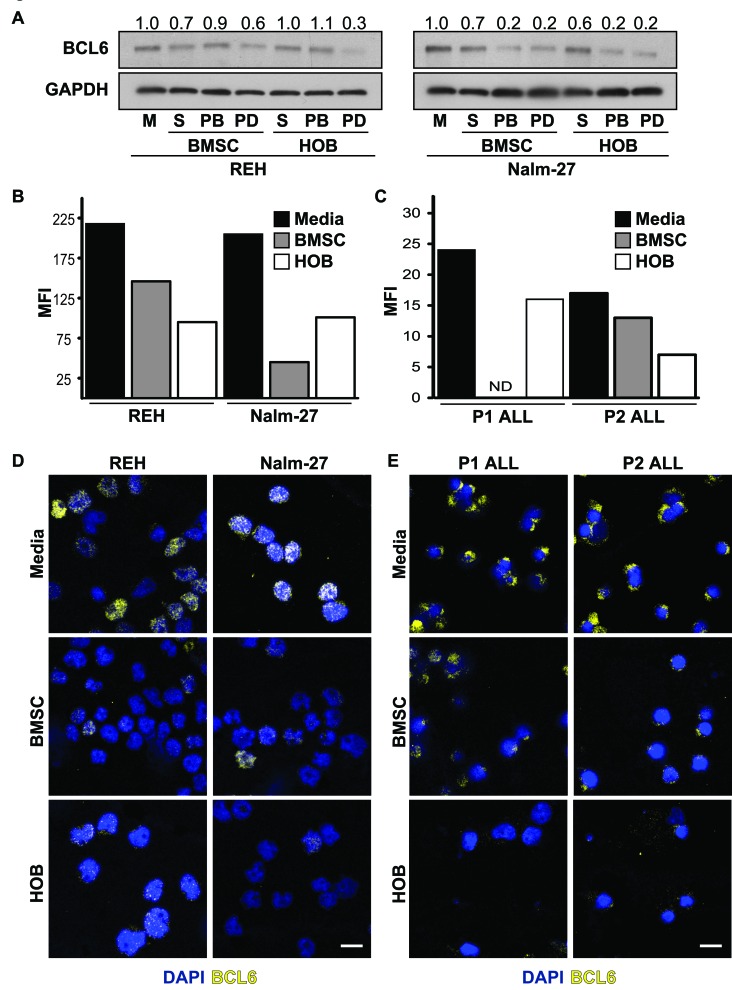
Co-culture with BMSC or HOB reduces BCL6 in ALL cells **A.** BCL6 protein in REH and Nalm-27 ALL cells when co-cultured with BMSC or HOB cells relative to media (M) controls as shown by western blot analysis. **B.** Flow cytometry analysis of REH and Nalm-27 ALL cell BCL6 protein levels when removed from the PD population compared to cells in media alone as shown by median florescence intensity (MFI). **C.** MFI of Patient 1 (P1) and Patient 2 (P2) when in physical contact with BMSC or HOB compared to those in media alone (ND = not detected). **D.** Confocal microscopy images of REH and Nalm-27 for BCL6 (yellow) and DAPI (Blue) in cells cultured in media alone compare to those recovered from the PD population of BMSC or HOB co-culture. **E.** P1 and P2 BCL6 confocal staining of media alone cells relative to those in contact with BMSC or HOB. Scale bar = 10μm.

Both the BMM in general, and BCL6 specifically, have independently been shown to regulate ALL survival [11- 14, 30, 31]. However, it has not been determined whether there is a functional link between bone marrow niche derived signals and ALL cell abundance of BCL6. To determine whether BMM cells regulate BCL6 protein levels in leukemic cells, ALL cell lines were grown in co-culture with either BMSC or HOB and compared to tumor cultured in media alone. Co-culture derived tumor cells were further sub-divided into distinct populations that included suspended (S), phase bright (PB), and phase dim (PD) leukemic cells based on their spatial location within the co-culture. We have previously observed that *in vitro* location related to BMSC or HOB stromal cells impacts ALL survival in co-culture during chemotherapy exposure, with the PD population of leukemic cells being the most resistant to chemotherapy exposure [13, 15] providing an opportunity to focus studies uniquely on the most resistant subpopulation of tumor cells. In the current study, regardless of the fraction of ALL cells evaluated, decreased BCL6 protein abundance was observed in ALL cells co-cultured with BMSC or HOB, with the most pronounced reduction consistently observed in the PD population (Figure 1A-1B). Of note, under normal culture conditions there is no difference in ALL cell viability between cells cultured in media alone compared to those in the co-culture conditions (DNS) supporting the observation that changes in BCL6 abundance are not due to selective pressure of the different culture conditions, but are a result of interactions with the BMSC or HOB. Consistent with western blot observations, flow cytometry and confocal microscopy analysis of REH and Nalm-27 cell lines showed that leukemic cells recovered from the PD population of BMSC or HOB co-culture had reduced BCL6 protein abundance compared to tumor cells cultured in media alone (Figure 1B, and 1D). Consistent with data derived from cell lines, ALL patient derived cells co-cultured with BMSC or HOB also had decreased BCL6 protein levels compared to cells grown in media alone (Figure 1C, and 1E).

### Modulation of BCL6 alters ALL cell cycle progression and proliferation rate

**Figure 2:**
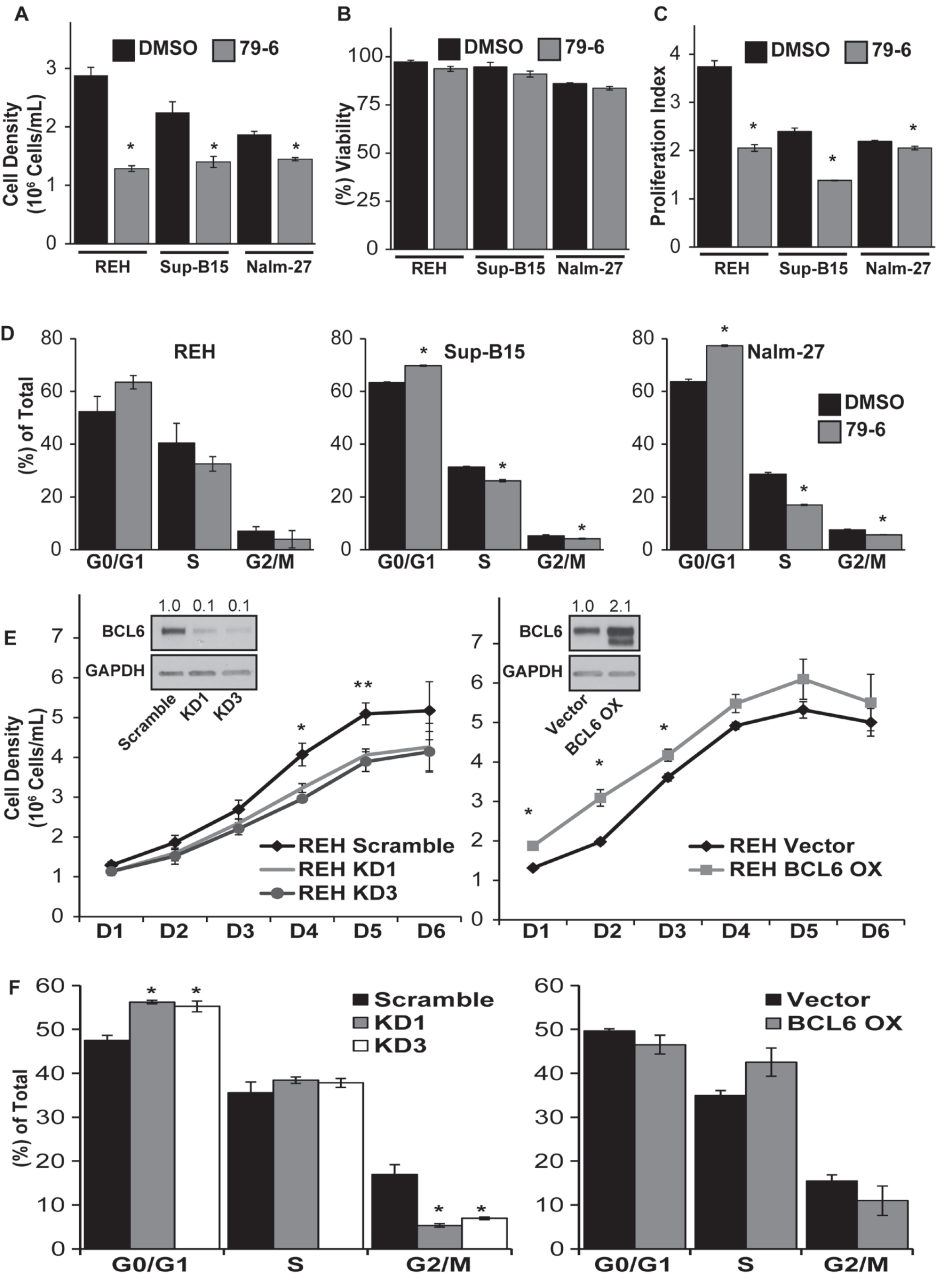
Modulation of BCL6 alters cell cycle progression and proliferation of ALL cells **A.**- **B.** Cell density and viability of REH, Sup-B15, and Nalm-27 following exposure to the small molecule BCL6 inhibitor 79-6 (125μM) relative to DMSO controls as shown by trypan blue exclusion cell counts. **C.** Proliferation index of 79-6 treatment of REH, Sup-B15 and Nalm-27 ALL cells compared to DMSO controls using a CSFE cell retention dye flow cytometry analysis. **D.** Propidium iodide (PI) DNA staining for cell cycle assessment of REH, Sup-B15 and Nalm-27 treated with 79-6 compared to DMSO controls. **E.** Cell density of shRNA knockdown of BCL6 (KD1 and KD3) (left panel) and BCL6 overexpression (BCL6 OX) (right panel) of REH cells over time compared to vector controls as evaluated by trypan blue exclusion counts. **F.** Cell cycle analysis of BCL6 knockdown (left panel) and BCL6 overexpression (right panel) in REH cells using PI staining. (* = *p* < 0.05 for 79-6 treated cells or knockdown/overexpression cells compared to DMSO or vector controls, respectively).

Based on reports of BCL6 abundance influencing proliferation of B-cells [32- 35], we determined the functional consequence of BCL6 downregulation on ALL cell proliferation and cell cycle progression. Inhibition of BCL6 with the small molecule inhibitor 79-6 resulted in a significant decrease in expansion of ALL cells in media alone compared to DMSO solvent controls (Figure 2A) without an effect on tumor cell viability (Figure 2B). Proliferation of ALL cells was reduced by BCL6 inhibition as reflected by a significant reduction in the proliferation index of ALL cells exposed to 79-6 (Figure 2C). Consistent with reduced ALL cell number and proliferation, BCL6 inhibition altered cell cycle progression in ALL cells as shown by an increase in G_0_/G_1_ phases and reduction in S and G2/M phases (Figure 2D). Because there is always concern regarding the potential for non-specific effects when using small molecule inhibitors, we generated lentiviral based shRNA knockdown of BCL6 in REH cells. This more specific targeted approach resulted in diminished proliferation as determined by a decrease in cell density over time relative to vector controls (Figure 2E; left panel). Conversely, overexpression of BCL6 in REH cells increased cell density compared to vector controls in a time course assay (Figure 2E; right panel). Knockdown of BCL6 also significantly increased the percentage of REH tumor cells in G_0_/G_1_ phases and reduced G_2_/M phases in line with the observed reduction of cell density in the time course assay (Figure 2F; left panel). Overexpression of BCL6 decreased the fraction of ALL cells in G_0_/G_1_ phases and increased tumor numbers in S phase (Figure 2F; right panel), although these changes were not statistically significant their trend is consistent with the cell density assay.

### BCL6 expression in ALL cells impacts abundance of cell cycle regulatory protein cyclin D3

**Figure 3:**
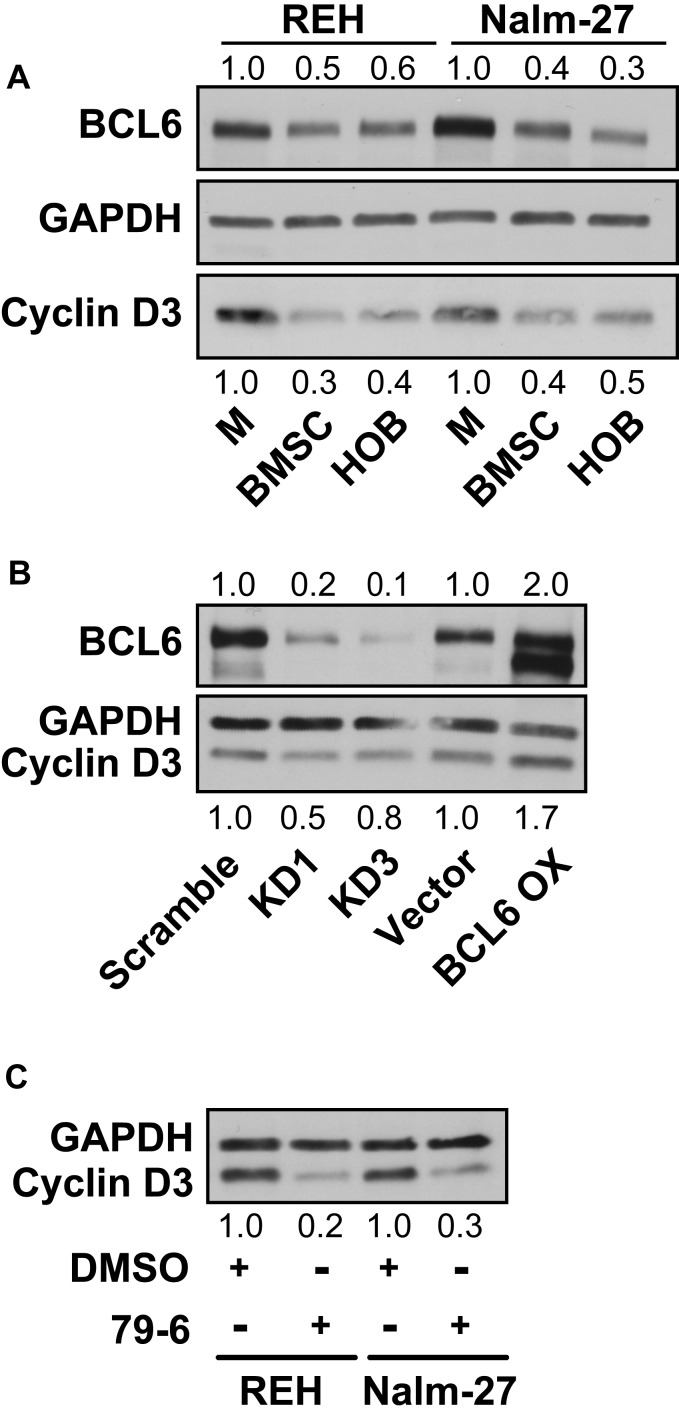
BCL6 modulates the cell cycle regulating protein cyclin D3 **A.** Western blot analysis of protein abundance of BCL6 and cyclin D3 in REH and Nalm-27 cells in media alone compared to PD cells recovered from BMSC or HOB co-culture. **B.** Comparison of REH BCL6 knockdown and overexpression to vector controls for BCL6 and cyclin D3 protein levels by western blot. **C.** Protein analysis by western blot of cyclin D3 in REH and Nalm-27 cells when exposed to 79-6.

Cyclin D3 has been shown to be an important cell cycle regulatory protein in germinal center B-cells, which is also a site where BCL6 is actively modulated to promote proliferation [36]. Based on these observations, we investigated whether BCL6 modulation impacts expression of cyclin D3. Consistent with BCL6 protein levels, cyclin D3 protein abundance was decreased in PD REH and Nalm-27 ALL cells compared to tumor cells grown in media alone (Figure 3A). Knockdown of BCL6 in ALL cells reduced the protein abundance of cyclin D3, and BCL6 overexpression increased cyclin D3 protein levels (Figure 3B). In addition, chemical inhibition of BCL6 by 79-6 led to diminished cyclin D3 protein abundance in ALL cells (Figure 3C).

### Chronic overexpression of BCL6 sensitizes the chemotherapy-resistant PD population to chemotherapy

**Figure 4:**
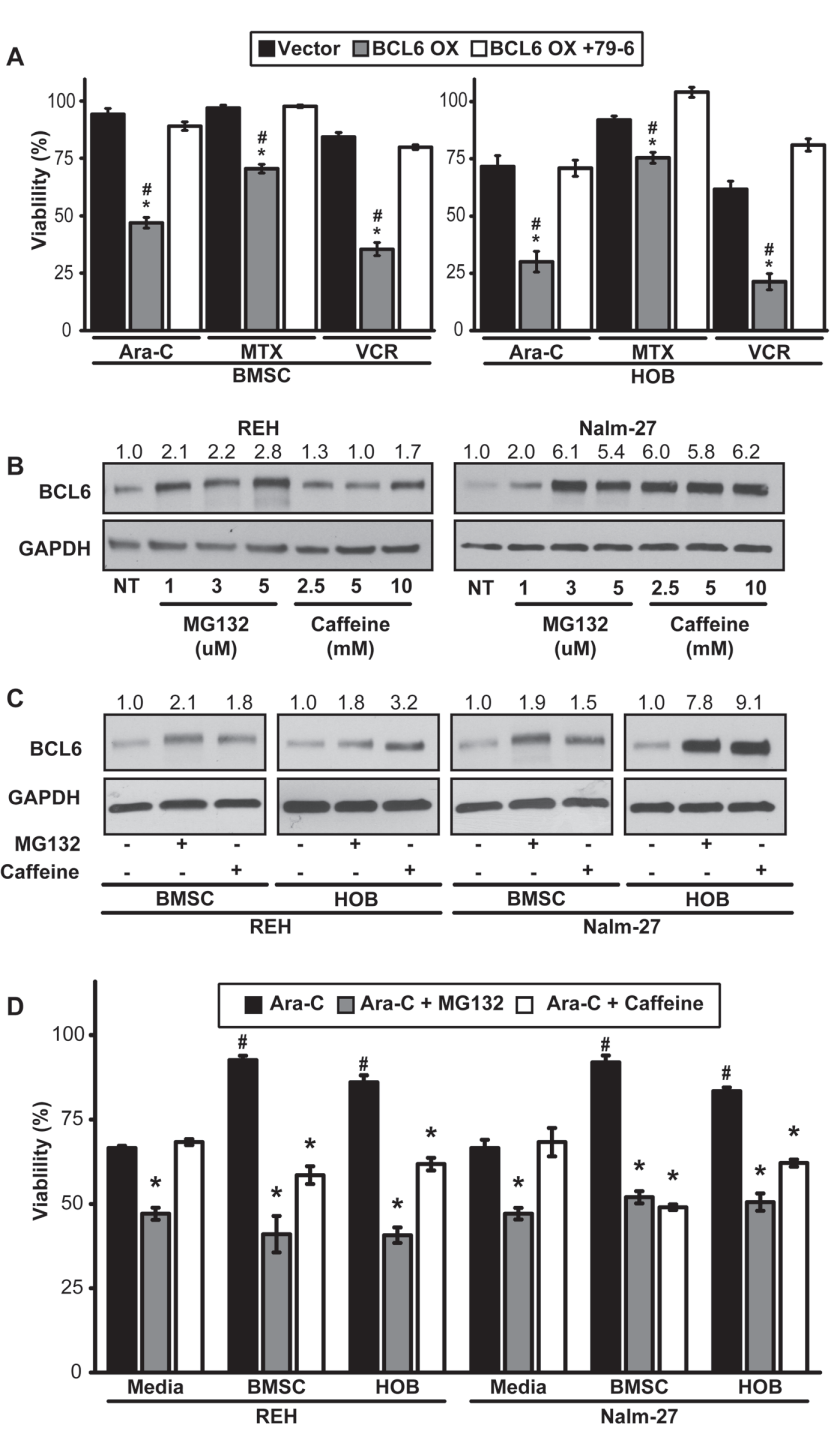
Forced expression of BCL6 sensitizes PD ALL cells to chemotherapy exposure **A.** Viability comparison of REH vector control, BCL6 overexpression, or BCL6 overexpression cells pre-treated with 79-6 (125μM) following exposure to three chemotherapy drugs (Ara-C [1 μM], MTX [50 μM], VCR [25 μM]). (* = *p* < 0.05 BCL6 OX to vector control and # = *p* < 0.05 BCL6 OX to BCL6 + 79-6). **B.** REH and Nalm-27 BCL6 protein dose response to MG132 and caffeine as shown by western blot. **C.** Western blot analysis to determine BCL6 protein abundance of REH and Nalm-27 cells when exposed to MG132 or caffeine when recovered from media alone, and the PD population of BMSC or HOB co-culture. **D.** REH and Nalm-27 cell viability following exposure to Ara-C alone or when pre-treated with MG132 or caffeine 6 hours prior to Ara-C exposure to compare cells in media alone to those recovered from the PD population of BMSC or HOB co-culture. (# = *p* < 0.05 PD ALL cells from BMSC/HOB relative to media and * = *p* < 0.05 Ara-C + MG132/Ara-C + caffeine relative to Ara-C only treatment).

Many ALL chemotherapy regimens rely on tumor cell proliferation as a requirement for optimal induction of cell death. Consequently, these strategies tend to be less effective against quiescent tumor cells [12, 37]. With the observation that reduced BCL6 in PD ALL cells results in a quiescent phenotype, we aimed to investigate strategies that target this chemotherapy-resistant population through modulation of BCL6. REH tumor cells with constitutive overexpression of BCL6 in the PD population showed a significant reduction in viability when compared to vector controls following exposure to chemotherapy (Figure 4A). PD tumor cells were “rescued” from BCL6 overexpression by BCL6 chemical inhibition, as demonstrated by the increase in PD REH cell viability following 79-6 and chemotherapy exposure relative to the overexpression only cells (Figure 4A). Based on this observation we identified chemical compounds that influence BCL6 protein levels. MG132 and caffeine have been shown to increase BCL6 protein abundance in cells by preventing the degradation of BCL6 [27]. While it is appreciated that neither MG132 or caffeine are specific regulators of BCL6, and that the effects of either could be on an upstream modulator of BCL6, our findings showed that MG132 or caffeine exposure resulted in increased BCL6 protein in ALL cells (Figure 4B). Given that PD cells have less BCL6 and are more resistant to chemotherapy, we investigated whether MG132 or caffeine exposure increased BCL6 in PD ALL cells. Exposure to either MG132 or caffeine increased BCL6 protein abundance in PD ALL cells (Figure 4C). Consistent with our previously published data [13, 15], PD ALL cells in both BMSC and HOB are protected from chemotherapy exposure relative to their media alone counterparts as indicated by significantly increased viability following Ara-C exposure (Figure 4D). However in both REH and Nalm-27 cells, pretreatment with MG132 or caffeine 6 hours prior to Ara-C exposure sensitized the resistant PD ALL cell population to chemotherapy-induced death as shown by a significant reduction in cell viability compared to the group treated with Ara-C alone (Figure 4D).

### Forced expression of BCL6 in ALL cells increases chemotherapeutic response

**Figure 5:**
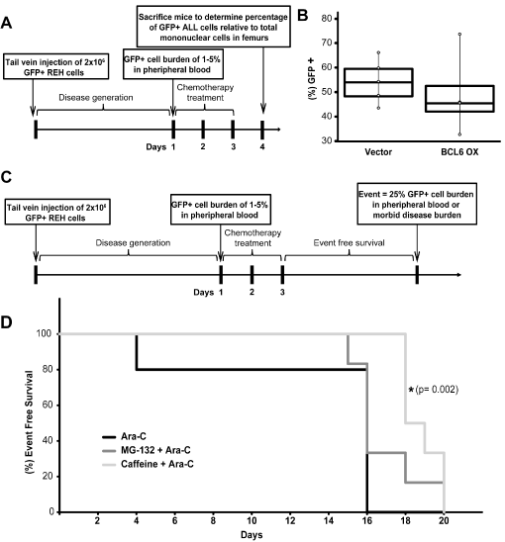
*In vivo* sensitivity to Ara-C is increased by BCL6 overexpression or pre-treatment with caffeine **A.** Schematic of NSG mouse experiment to determine GFP+ ALL burden in the femurs of NSG mice. **B.** Box plot representation of median percentage of GFP+ REH ALL cells relative to total mononuclear cells recovered from femurs of NOD-SCID Gamma (NSG) mice infected with REH vector control (*n* = 5) or REH BCL6 overexpression (*n* = 4) ALL cells following three consecutive days of Ara-C treatment. **C.** Schematic of NSG mouse experiment to determine event free survival of mice pre-treated with BCL6 modulating drugs MG-132 or caffeine. **D.** Event free survival of NSG mice following treatment with Ara-C (*n* = 5), MG-132 + Ara-C (*n* = 6), or caffeine + Ara-C (*n* = 6) (* = *p* < 0.05 Ara-C relative to caffeine+ Ara-C).

Residual tumor cells in the bone marrow following chemotherapy treatment is a prognostic indicator of patient outcome [4- 6]. Based this well-established indicator we evaluated tumor burden in the bone marrow of NOD-SCID gamma (NSG) mice following treatment with chemotherapy (Figure 5A). Although not statistically significant mice injected with ALL cells overexpressing BCL6 had a lower median percentage (45.6% GFP+) of human tumor cells compared to those injected with vector control cells (54.1% GFP+) 24 hours after the conclusion of Ara-C treatment (Figure 5B). Because MG132 and caffeine sensitized the chemotherapy-resistant PD ALL cells to chemotherapy *in vitro* (Figure 4D), we investigated whether MG132 or caffeine could increase event free survival in a NSG model of ALL disease (Figure 5C). Corresponding to the *in vitro* observations, mice pretreated with caffeine 6 hours prior to Ara-C treatment had significantly increased event free survival time compared to mice treated with Ara-C only (Figure 5D).

## DISCUSSION

In the current study, we investigated the role that bone marrow stromal cells and osteoblasts have on the modulation of BCL6 levels in ALL, and the influence of BCL6 on resistance to chemotherapy. While there are numerous established BMM interactions that regulate ALL proliferation and chemotherapy resistance, to our knowledge this work represents the first time microenvironment regulation of ALL BCL6 abundance has been explored. Utilizing BMSC and HOB as just two representative elements of the protective BMM niche, we observed that co-culture reduces tumor cell BCL6 expression compared to ALL cells cultured in media alone (Figure 1) and that removal of ALL cells from the PD “niche” buried beneath BMSC or HOB to media alone results in increased BCL6 protein abundance (DNS). The reduction of BCL6 in ALL cells that are in co-culture with bone marrow derived adherent stromal cells or osteoblasts is most pronounced in the PD sub-population of ALL cells, which we have previously reported as the most quiescent and refractory to chemotherapy [13, 15]. The quiescent phenotype appears to be regulated, in part, through BCL6 impact on ALL cell cycle progression. Both chemical inhibition and targeted knockdown of BCL6 in ALL cells resulted in diminished proliferation and accumulation of cells in the G_0_/G_1_ phase of cell cycle (Figure 2). Likewise, increased abundance of BCL6 led to sustained proliferation and a higher percentage of cells in S phase (Figure 2) when compared to vector control cells. The ability of BCL6 to regulate the transition of cells between quiescent and proliferative states is reminiscent of its function in germinal center B-cells where elevated BCL6 acts to promote high rates of proliferation [33- 35]. Consistent with the broadly recognized complexity of the impact of BCL6 on cell fate, it has been shown that, in contrast to our findings, BCL6 upregulation in some settings promotes a quiescent phenotype [38- 40]. These differences are not surprising as BCL6 is known to interact with, and regulate, a variety of cellular programs in a context dependent manner [reviewed 41- 43] and highlights the importance of investigating BCL6 in the specific setting of microenvironment regulation and to interpret observations with the appropriate model driven limitations in mind. In the current study, we show that BCL6 influences proliferation of ALL cells and that its abundance is influenced by the interaction with elements of the BMM (Figure 1 and 2). In addition, our observations suggest that cyclin D3 protein levels in ALL cells are, in part, regulated by BCL6. Both chemical inhibition and more specific shRNA knockdown of BCL6 in ALL cells reduced cyclin D3 levels with BCL6 overexpression correlated with increased cyclin D3 protein abundance (Figure 3). This observation is significant as cyclin D3 has been reported to be an important regulator of mature and immature B-cell cell cycle progression through G_1_ phase [36, 44, 45]. While the precise mechanism by which the BMM is regulating BCL6 abundance in ALL cells remains unknown, one possibility that warrants consideration is that BCL6 protein being regulated *via* niche derived cues that impact on phosphorylation, targeting it for proteasomal degradation. Based on previously described pathways that regulate BCL6 [27, 46, 47] and our observations using proteasome inhibitors (Figure 4), as well as, the lack of significant change in BCL6 mRNA levels in tumor cells co-cultured with BMSC or HOB (DNS), regulation at the protein level is implicated. Future work which focuses investigation on this potential mechanism will be important, however this is beyond the scope of the current study. While additional studies will be required to focus on a greater understanding of the interactions between the BMM and ALL cells that drive the reduction in BCL6, our results suggest that the quiescent phenotype exhibited by ALL cells in the BMM niche is in part modulated through microenvironment regulation of ALL cell BCL6 protein. This in turn appears to regulate cell cycle progression, potentially through control of cyclin D3.

In both normal and malignant B-cells, increased expression of BCL6 has been shown to promote cell survival through inhibition of the p53 pathway, which allows for tolerance to DNA damage within cells [20, 30, 31]. In ALL cells, increased expression of BCL6 results in a tolerance to DNA damage and subsequently increased survival during BCR-ABL1 kinase inhibition [30]. Conversely, our observations suggest that decreased abundance of BCL6 subsequent to interaction of leukemic cells with BMSC or HOB can also protect ALL cells from death through induction of a quiescent phenotype. Furthermore, chronic overexpression of BCL6 appears to sensitize tumor cells to chemotherapy exposure coincident with increased ALL cell proliferation and blunted tumor cell quiescence (Figures 2 and 4). We speculate based on the work of others, as well as these observations that dynamic regulation of BCL6 in ALL regulates survival when challenged by stress such as chemotherapy. These observations suggest that increased BCL6 protein levels during chemotherapy may allow tolerance of DNA damage, with subsequent downregulation of BCL6 required for cells to enter a quiescent state during which DNA can be repaired. Interference of this dynamic balance, such as that imposed by chronic sustained expression of BCL6, appears one way in which to sensitize BMM protected ALL cells to chemotherapy treatment (Figures 4-5). Due to the complexities of both BMM signaling and BCL6 regulation, additional studies will be needed to determine how these dynamic regulatory pathways affect survival pathways including p53, ATM/ATR, and BCL family proteins within ALL cells and how this may promote resistant disease in the marrow niche.

Consistent with the *in vitro* findings, *in vivo* chronic overexpression of BCL6 during Ara-C treatment resulted in a modest reduction in the tumor burden in femurs of mice when collected 24 hours following the conclusion of Ara-C treatment (Figure 5B). In addition, using a model based on that which was previously described with the readout of event free survival [48- 50], we observed that caffeine pre-treatment, shown to increase BCL6 [27], significantly extended event free survival in a NSG mouse model of ALL (Figure 5D). While recognizing that caffeine does not specifically target BCL6 exclusively, it may serve as a safe tool to, at least in part, modulate BCL6 expression. Diminished tumor burden in the bone marrow and event free survival have both been shown to be significant prognostic indicators of patient outcome in response to chemotherapy [5, 7, 51] and these findings illustrate the significance of the observed increase in event free survival time of mice following combination treatment with caffeine and Ara-C. We also hypothesize that this type of combination treatment strategy might be advantageous during consolidation therapy as a means to “activate” residual quiescent ALL cells to be better targeted by cytotoxic regimens. In this context, caffeine is an attractive treatment strategy due to its long history of safe use in humans [52] and our results which show it can sensitize microenvironment protected ALL cells to chemotherapy treatment (Figures 4-5). As with all models in immunocompromised mice there are limitations to interpretation, however, they serve as an important setting in which to test general concepts and to identify potentially important pathways around which to focus novel intervention strategies.

In summary, the goal of this study was to investigate how BMSC and HOB, components of the protective bone marrow niche, would influence the levels of BCL6 in ALL cells. We report that ALL cell lines, as well as primary patient samples, co-cultured with BMSC or HOB, have reduced BCL6 protein. This reduction in BCL6 abundance was most pronounced and consistently observed in leukemic cells recovered from the PD population, which we have previously characterized as a chemotherapy-resistant population representative of resistant tumor populations [13, 15]. Decreased BCL6 in ALL cells affects the cell cycle profile and promotes a quiescent phenotype. This phenotype appears to be coincident with BCL6 reduction and decreased cyclin D3; a consequence that has been reported to regulate progression through the G_1_ phase of cell cycle [36, 44, 45]. Chronic overexpression of BCL6, achieved either through overexpression vectors or chemical intervention by MG132 or caffeine, sensitized ALL cells that are generally protected by BMSC or HOB from chemotherapy induced death. Furthermore, combination treatments using caffeine to stabilize BCL6 levels followed by Ara-C exposure significantly increased the event free survival of mice in which ALL had been established. Collectively, these results suggest that strategies which disrupt microenvironmental regulation of BCL6 in ALL cells may be an effective strategy to sensitize quiescent, chemotherapy-resistant leukemic cells to treatment, eliminating MRD in the protective bone marrow niches and reducing the incidence of relapse.

## MATERIALS AND METHODS

### Cell lines and culture conditions

Philadelphia chromosome positive (Ph+) lymphoblastic cell lines Nalm-27 (Fujisaki Cancer Center) and Sup-B15 (ATCC-CRL-1929), and Ph- REH (ATCC#CRL-8286) were utilized. De-identified primary human leukemic cells were acquired from the West Virginia University Health Sciences Center and West Virginia University Cancer Institute tissue bank. Primary patient sample 1 (P1) is a MLL rearranged (11q23) B-lineage ALL isolated from a 43 year old female at diagnosis. Primary patient sample 2 (P2) is a (Ph^−^) B-cell ALL/LBL isolated from a 65 year old male at diagnosis (45-46, XY, t(4-11)(q21;q23), add (6)(p25), −21, +1-2mar[12]/46, XY[8]). De-identified primary bone marrow stromal cells (BMSC) were provided by the West Virginia University Cancer Institute Biospecimen Processing Core and the West Virginia University Department of Pathology Tissue Bank. BMSC cultures were established as previously described [53]. Human osteoblasts (HOB; PromoCell) were cultured according to the supplier's recommendations. Co-cultures of adherent bone marrow derived supportive cells and ALL cells were established by seeding leukemic cells onto 80-90% confluent BMSC or HOB monolayers. Cultures were fed every 4 days and tumor cells collected for inclusion in experiments. Remaining leukemic cells were moved to new primary BMSC or HOB adherent layers every 12 days. Cultures were maintained in 5% O_2_ to model normal bone marrow oxygen tension, reported to range from 1-7% [54, 55]. Suspended (S) leukemic cells floating freely in the media; phase bright (PB) tumor cells, that were loosely adherent to the top of BMSC or HOB; and phase dim (PD) leukemic cells that were buried firmly beneath adherent BMSC or HOB were collected as distinct populations as previously described [13, 15]. The S, PB, and PD tumor populations were separated from BMSC or HOB by size exclusion with G10 Sephadex (Sigma) column separation as previously described [13, 15, 56].

### Flow cytometric quantification of BCL6 expression

REH and Nalm-27 tumor cells were cultured and PD ALL cells were harvested as described above. P1 and P2 were cultured in media alone or co-cultured with BMSC or HOB for 2 days prior to analysis to utilize them prior to significant loss in viability. Experiments that included primary tumor cells required collection of all tumor that was in physical contact with the BMSC or HOB (PB + PD) to provide sufficient numbers for analysis. ALL cells were stained using Cell Signaling Technology's recommended protocol for intracellular BCL6 staining using primary antibodies rabbit anti-BCL6 (Cat # 14895) (1:300) or Rabbit (DA1E) mAb IgG XP isotype control (Cat # 3900). Cells were washed with 1x PBS and incubated with secondary antibody goat anti-rabbit Alexa Flour 647 (Invitrogen; Cat # A21244) [1 μg/mL]. Collection and analysis were performed using the LSRFortessa (Becton Dickenson, San Jose, CA, USA).

### Immunofluorescence imaging

Confocal images were acquired using an upright LSM 510 Zeiss microscope and processed using Zen2009 software and Adobe Photoshop with fluorescence intensity held constant for any experiment in which image acquisition was compared across samples. ALL cells were cytospun on glass slides following G10 Sephadex purification. Cells were fixed with 4% PFA, blocked in 1x PBS/5%FBS/0.3% Triton X-100, washed with 1x PBS, and incubated with rabbit anti-BCL6 (Cell Signaling Technology, Cat # 14895) (1:100) followed by anti-rabbit Alexa 647 (Invitrogen; Cat # A21244) (1:200). Slides were washed with PBS and mounted to coverslips using Prolong^®^ Gold anti-fade/DAPI overnight (Life Technologies).

### Cell proliferation assay

ALL cells were labeled using the cell retention dye CellTrace-CFSE Cell Proliferation Kit (Life Technologies, Cat # C34554) as described by the manufacturer. Cells were then cultured under normal growth conditions for 2 days in either media DMSO control or media with 79-6. CellTrace fluorescence intensity was measured by flow cytometry using FACSFortessia. Proliferation indices were calculated using FCS Express4.

### Cell cycle analysis

ALL cells were fixed in 70% ethanol, treated with RNase (Sigma), and stained with propidium iodide (PI) for DNA analysis. All samples were performed in triplicate, processed on a FACSFortessia flow cytometer and analyzed using FCS Express4 software.

### Western blot analysis

Rabbit polyclonal BCL6 (Cat # 5650) and Cyclin D3 (Cat # 2936) were purchased from Cell Signaling Technology and used at 1:1000 dilution. Mouse polyclonal anti-GAPDH was purchased from Fitzgerald Inc. (Cat # 10R-G109a). Proteins were resolved on SDS-PAGE gels and transferred to nitrocellulose membranes. Membranes were blocked in TBS 5%/nonfat dry milk 0.05% Tween-20 and probed with the indicated primary antibodies. After incubation with horseradish peroxidase-conjugated secondary antibodies, signal was visualized using enhanced chemiluminescence reagents (Amersham). Western blots are representative of at least 3 independent experiments. Densitometry quantification is indicated and was completed using ImageJ software.

### Drugs and chemotherapeutic reagents

Cytarabine (Ara-C) (Selleckchem, Cat # S1648), Methotrexate (MTX) (Selleckchem, Cat # S1210), Vincristine (VCR) (Selleckchem, Cat # S1241), MG132 (Selleckchem, cat # S2619), Caffeine (Sigma-Aldrich, Cat # C0750), and 79-6 (Calbiochem, Cat # 197345) were diluted and stored per manufacturer recommendations. For *in vitro* experiments drug stocks were diluted in base media and for *in vivo* experiments stocks were diluted in saline immediately prior to use. *In vitro* concentrations of Ara-C [1 μM], MTX [50 μM], VCR [25 μM], MG-132 [1-5 μM], caffeine [2.5-10 mM], and 79-6 [125 μM] were used to approximate clinically relevant doses in ALL or published *in vitro* concentrations [27, 57- 63].

### Evaluation of leukemic cell concentration and viability

ALL cells were cultured in media alone or co-cultured with BMSC or HOB for 4 days to establish the PD tumor population. On day 4 cultures were provided fresh media and exposed to Ara-C, MTX, or VCR for 4 additional days. Cells treated with Ara-C were re-treated at 48 hours. 79-6, MG132, or caffeine were added 6 hours prior to chemotherapy in combination experiments. Viability and cell number were evaluated by trypan blue exclusion in triplicate.

### BCL6 knockdown and overexpression

Human TRIPZ lentiviral inducible shRNAmir constructs to BCL6 clone ID numbers V3THs_404721 (KD1) and V2THS_132926 (KD3) were purchased from Thermo Scientific. Viral particles were produced and administered to REH ALL cells according to manufactures protocol. shRNA expression was induced using doxycycline [1ug/mL] and RFP positive cells were sorted by flow cytometry.

BCL6 overexpression vector was generated by removing the BCL6 gene sequence from the MSCV-BCL6-IRES-GFP [40] which was purchased from Addgene (Plasmid 31391). BCL6 fragment was then ligated into pLVX-EF1α-IRES-ZsGreen1 plasmid (Clontech Laboratories, Inc. Cat# 631982).

### Mice

All experimental procedures involving NOD/SCID Gamma (NSG) mice were approved by the West Virginia University Institutional Animal Care and Use Committee. Male NOD/SCID Gamma (NSG) mice age 5-6 weeks were acquired from the West Virginia University NSG colony or purchased from the Jackson Laboratory. To determine whether chronic BCL6 overexpression would sensitize ALL cells to chemotherapy treatment, resulting in reduced tumor burden in the bone marrow, NSG mice were divided into two groups and tail vein injected with 2 ×10^6^ REH cells expressing BCL6/GFP or vector/GFP control. Peripheral tumor burden was monitored *via* tail vein draws to collect approximately 30μL of blood. Red blood cells were lysed (150 mM NH_4_Cl, 10 mM NaHCO_3_ and 0.1 mM EDTA in distilled water) and ALL cell frequency was evaluated by flow cytometry analysis of GFP positive human cells relative to total mononuclear cells. Chemotherapy treatment began when the peripheral blood burden of the group reached 1-5% GFP positive cells which has been previously reported to indicate established leukemic disease [48]. Ara-C treatment was administered by intraperitoneal (IP) injection at a concentration of 100 mg/kg daily for 3 consecutive days. Mice were euthanized 24 hours after the final Ara-C treatment and bone marrow was collected from femurs to quantify percentage of GFP positive tumor cells in the bone marrow by flow cytometry.

To determine whether combination treatment of mice with the BCL6 modulating agents MG132 or caffeine would sensitize ALL cells to chemotherapy, 2×10^6^ REH cells expressing the vector/GFP construct were injected *via* tail vein to establish leukemic disease. Tumor burden was monitored as previously described. When peripheral blood burden of the group reached an average of 1-5% GFP positive cells relative to total mononuclear cells, mice were randomly assigned to treatment groups. Treatments included saline control, MG132 [1 mg/kg], Caffeine [50 mg/kg], Ara-C [100 mg/kg], MG132 [1mg/kg] + Ara-C [100 mg/kg], or Caffeine [50 mg/kg] + Ara-C [100 mg/kg]. All drugs were diluted in saline prior to injection and were administered by IP injection. MG132 and caffeine were given 6 hours before treatment with Ara-C with mice treated for 3 consecutive days. Event free survival (EFS) was calculated from the start of treatment as previously described [48] with an event defined as 25% GFP positive cells in the peripheral blood by flow cytometric analysis or when mice showed clinical signs of disease (lethargy, weight loss, ruffled fur).

### Statistical analysis

All data are presented as mean ± standard error and the statistical significances between conditions was determined by the student's t test or 2-way ANOVA with Holm-Sidak post-hoc test using GraphPad or SigmaPlot software. All *in vitro* results generated from cell line derived data are representative of at least 3 independent experiments. Experiments with primary patient samples are representative of at least 2 independent experiments. Kaplan-Meier survival curves were generated for event free survival and a fitted Cox model was used to determine p-values.
